# Granulocytic sarcoma of cervix: A rare case report

**DOI:** 10.1097/MD.0000000000029419

**Published:** 2022-06-17

**Authors:** Zhongxue Ye, Yafen Jiang

**Affiliations:** Department of Gynecology, Hwa Mei Hospital, University of Chinese Academy of Sciences, Ningbo, China.

**Keywords:** granulocytic sarcoma, cervix, chemotherapy, acute myeloid leukemia, case report

## Abstract

**Rationale:**

Granulocytic sarcoma (GS) is an uncommon extramedullary tumor, and involvement of the female reproductive system is very rare.

**Patient concerns:**

We present a case of cervical GS in a 45-year-old woman who presented with repeated vaginal bleeding after sex for 1 month.

**Diagnosis:**

The patient was diagnosed with cervical GS mainly based on pathological immunohistochemical examination and further progressed to acute myeloid leukemia (AML) based on bone marrow puncture and cytogenetic analysis.

**Interventions and outcomes:**

The patient underwent hysterectomy and bilateral adnexectomy, and subsequently received AML-type chemotherapy. She relapsed 3 months after therapy and progressed to AML. The patient was then treated with chemotherapy with cytosine arabinoside and idarubicin again and achieved complete remission after 1 cycle. Currently, she is still receiving therapy combined with cytosine arabinoside and idarubicin, and has been alive for 13 months.

**Lessons:**

Although GS of the reproductive system is rare, it should be included in the differential diagnosis of gynecological neoplasms and should be treated with AML-type chemotherapy protocols.

## 1 Introduction

Granulocytic sarcoma (GS) is a rare extramedullary tumor consisting of immature myeloid cells. It is often found simultaneously with acute leukemia or less commonly with myelodysplastic syndrome, chronic myeloid leukemia, and other myeloproliferative diseases.^[1]^ In rare cases, they appear as an isolated mass without prior evidence of hematological disease, which is called isolated or primary GS. The most common sites include lymphoid tissues, skin, bone, kidney, and the gastrointestinal tract.^[2,3]^ GS of the female reproductive system is rare. Here, we reported a case of cervical granulocytic sarcoma admitted to the Ningbo Hwa Mei Hospital, University of Chinese Academy of Science in February 2021. The patient was diagnosed with primary cervical GS with insufficient diagnosis of acute myeloid leukemia (AML). However, she relapsed rapidly and progressed to GS with AML during the follow-up.

## 2 Case report

**Figure 1:**
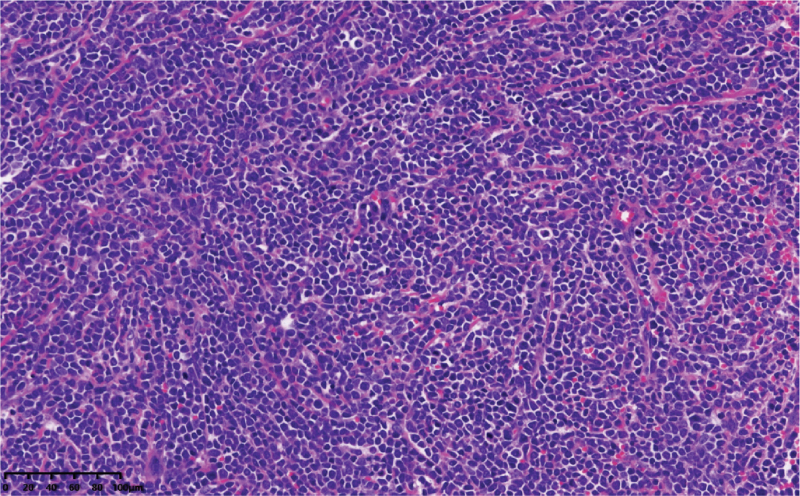
Cervical specimen showed infiltration by medium-size neoplastic cells with irregular nuclear contours (H&E, ×400).

**Figure 2:**
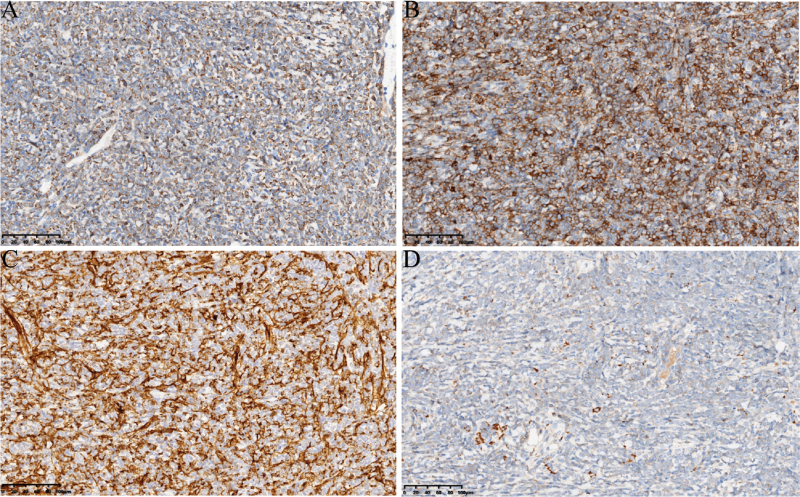
Tumor cells were positive for MPO (A), CD117 (B), CD34 (C) and CD68 (patrial, D) (×400).

A 45-year-old woman (gravida 2, para 1) experienced repeated vaginal bleeding after sex for 1 month. The patient had no significant medical or family history. The HPV test results were negative. Contrast-enhanced magnetic resonance imaging suggested abnormal changes in the posterior wall of the cervix, which were considered malignant. No lymph node enlargement was observed. Cervical biopsy showed granulocytic sarcoma (Fig. 1), with positive staining for MPO, CD117, CD99, CD34, and CD68 (partial) (Fig. 2), and negative staining for CD20, CD79a, CD10, CD3, CD56, CD138, desmin, CK, myogenin, EMA, and cyclin D1. Ki67 was expressed in 60% of the cells. The patient was admitted to our department on February 1, 2021. She provided written informed consent to publish this case report. Gynecologic examination revealed an enlarged cervix, approximately 5 cm in diameter, of which the lower lip was irregularly nodular and bruised easily without parametrial or vaginal involvement. Bone marrow smears revealed 1% primitive cells, and a bone marrow biopsy showed no significant abnormalities. Analysis of immunophenotype by flow cytometry suggested 3.1% abnormal myeloid primitive cells. Other laboratory tests, chest CT, and abdominal ultrasonography were normal.

The patient was diagnosed with cervical GS and underwent hysterectomy and bilateral adnexectomy on February 10, 2021. The surgical specimen displayed granulocytic sarcoma of the cervix sized 5 × 2 × 1.2 cm, infiltrating the deep fibrous layer of the cervix (more than 2/3 of the whole cervix), with vascular cancer embolus and nerve infiltration, but without parametrial involvement. Pathological findings also revealed the involvement of the vaginal fornix. The patient began chemotherapy with cytosine arabinoside (Ara-C) (100 mg/m^2^/d for 7 days) and idarubicin (IDA) (10 mg/m^2^/d for 7 days) 16 days after surgery. The analysis of immunophenotype by flow cytometry showed no significant abnormalities after 4 cycles (3 cycles of Ara-C and IDA, 1 cycle of Ara-C, and homoharringtonine). During this period, she experienced severe myelosuppression, which was resolved with symptomatic treatment. She refused chemotherapy after receiving Ara-C monotherapy again and was routinely followed up at the outpatient clinic. Three months after chemotherapy, the patient felt soreness of both lower extremities, and bone marrow smears suggested 25% abnormal myeloid primitive cells, which were involved in disease progression. The results of immunophenotype by flow cytometry also supported the diagnosis of AML, which reminded 24.03% of abnormal myeloid primitive cells. Chromosome analysis was performed, and the results of 45, X, -X, t (1; 4) (q21; q25)2/46, XX18 were reported. The patient was then treated with chemotherapy for Ara-C and IDA. Bone marrow smears and chromosomal examination suggested complete remission after 1 cycle. Currently, she is still receiving therapy combined with Ara-C and IDA.

## 3 Discussion

Granulocytic sarcoma, also known as chloroma because of its green color, caused by the presence of MPO, was first reported in 1823.^[4]^ The incidence of GS is 2 per million in adults and is 2.5% to 9.1% in patients with AML.^[2]^ Isolated GS is rare and patients usually develop to AML within several days or months. GS occurs at nearly any site; therefore, the clinical manifestations vary. The female reproductive system is less involved, and it was reported that the cervix was the most involved organ, estimated at 40.0% (22/54) in Chinese patients, followed by the ovaries (23.6%,13/55).^[5]^ In our case, the patient was diagnosed with cervical GS with mild abnormalities in her bone marrow test, which was not sufficient to diagnose AML. However, the patient relapsed rapidly and progressed to GS with AML during the follow-up.

The diagnosis of granulocytic sarcoma is relatively difficult, especially in the absence of leukemia. Most of them are poorly differentiated and are misdiagnosed as malignant lymphoma, Ewing sarcoma, poorly differentiated sarcoma, or small cell carcinoma.^[6]^ The Immunohistochemical stains is important for differential diagnosis. The most common positive markers in paraffin sections include MPO, CD68/KP1, CD117, CD99, CD 68/PG-M1, lysozyme, CD34, TdT, CD56, CD61, CD30, and glycophorin.^[7]^ Meanwhile, bone marrow aspiration, biopsy, and cytogenetic analysis are equally significant. This case was diagnosed as cervical GS mainly based on immunohistochemical examination, and further progressed to AML based on bone marrow puncture and cytogenetic analysis.

Owing to the rarity of GS, no treatment schemes are available based on prospective clinical trials. Data on treatment options come from case reports and small case series, most of which are retrospective studies. The treatment options include systemic chemotherapy, local radiotherapy, surgery, and allogeneic or autologous bone marrow transplantation.^[8–10]^ In view of a higher rate of progression to AML in isolated GS patients receiving localized treatment (88%-100%) compared with patients administered systemic chemotherapy (42%), it appears appropriate to treat GS with AML-type chemotherapy protocols regardless of AML status.^[6,11–13]^ Chemotherapy plus surgery (34.5%) was reported to be the most common option in Chinese patients with gynecological GS.^[5]^ In our case, the patient underwent hysterectomy, bilateral adnexectomy, and AML-type chemotherapy. Regrettably, she relapsed 3 months after surgery and chemotherapy and developed to AML.

The prognosis for GS is poor, with a 5-year survival rate of approximately 20%.^[7]^ It has been reported that the median survival for GS patients with or without AML is 6 to 14 months and 36 months, respectively.^[6]^ Our patient has been alive for 13 months and with stable disease currently.

In general, we herein reported a rare case of cervical GS that progressed to AML 3 months after the initial treatment. Gynecological GS should be considered in the differential diagnosis of gynecological neoplasms. Pathological immunohistochemical and bone marrow examinations are important for diagnosis. Despite its limited distribution, patients should be treated aggressively with chemotherapy.

## Author contributions

**Conceptualization:** Zhongxue Ye.

**Data curation:** Zhongxue Ye.

**Formal analysis:** Zhongxue Ye.

**Supervision:** Yafen Jiang.

**Writing – original draft:** Zhongxue Ye.

**Writing – review & editing:** Yafen Jiang.
